# Clinical impact of multidisciplinary carbapenem stewardship interventions: a retrospective cohort study

**DOI:** 10.1186/s40545-023-00599-0

**Published:** 2023-07-24

**Authors:** Anitha Ramadas, Rahela Ambaras Khan, Khairil Erwan Khalid, Chee Loon Leong, Mohd Makmor-Bakry

**Affiliations:** 1 Department of Pharmacy, Hospital Kuala Lumpur, Ministry of Health, Kuala Lumpur, Malaysia; 2 Faculty of Pharmacy, Universiti Kebangsaan Malaysia, Kuala Lumpur, Malaysia; 3 Infectious Diseases Unit, Department of Medicine, Hospital Kuala Lumpur, Kuala Lumpur, Malaysia

**Keywords:** Antimicrobial stewardship, Carbapenem, Mortality outcome, Clinical outcome, Antibiotics

## Abstract

**Background:**

Antimicrobial stewardship (AMS) program aims to optimise antimicrobial utilisation and curb antimicrobial resistance. We investigated the clinical impact of AMS among patients with carbapenem in medical wards of a tertiary hospital.

**Methods:**

A retrospective cohort study was conducted on hospitalised adult patients treated with carbapenem and reviewed by a multidisciplinary AMS team. We compared the clinical outcomes of accepted (n = 103) and not-accepted AMS intervention cases (n = 37). The outcomes evaluated include trends of total white blood cells (TWBC), C-reactive protein (CRP), body temperature at day-7, and clinical status at day-30 post-AMS intervention.

**Results:**

The interventions included discontinuation (50%), de-escalation (47.9%) and escalation (2.1%) of antibiotics, where the acceptance rate was 67.1%, 80.6% and 66.7%, respectively. Overall, we found no significant difference in clinical outcomes between accepted and not-accepted AMS interventions at day-7 and day-30 post-interventions. On day-7, 62.0% of patients in the accepted group showed decreased or normalised TWBC and CRP levels compared to 47.4% of the not-accepted group (p = 0.271). The mortality at day-30 (32% *versus* 35%, p = 0.73), discharge rate (53.4% *versus* 45.9%, p = 0.437), and median length of hospital stay (36.0 *versus* 30.0 days, p = 0.526) between the groups were comparable. The predictors of 30-day mortality in the study subjects were Charlson Comorbidity Index > 3 (OR: 2.84, 95% CI 1.28–6.29, p = 0.010) and being febrile at day-7 (OR: 4.58, 95% CI 1.83–11.5, p = 0.001).

**Conclusion:**

AMS interventions do not result in significant adverse clinical impact and mortality risk.

## Background

World Health Organization (WHO) 2014 report on global antimicrobial resistance surveillance revealed that antibiotic resistance is a severe health threat worldwide [1]. The report recognised non-judicious antimicrobial consumption as the key driver in the development of bacterial resistance. This is a critical issue for low- and middle-income countries, including Malaysia. Malaysia witnessed an increase in antimicrobial consumption; the total utilisation of antibiotics increased by 7.3% in 2018 compared to 2017, and carbapenem was one of the most commonly used antibiotic classes [2, 3]. Healthcare-associated infections caused by multi-drug resistant organisms (MRO), particularly the carbapenem-resistant Enterobacterales (CRE), are on the rise and pose a significant concern [4].

Optimisation of antimicrobial medicines usage through antimicrobial stewardship (AMS) is one of the key strategies recommended by the WHO to prevent antimicrobial resistance [5]. The AMS has been defined as a coordinated intervention to improve the appropriate use of antimicrobial agents by promoting the optimal antimicrobial drug regimen, namely selection of agent, dosing, duration of therapy, and route of administration [6]. Evidence supports the impact of AMS on decreasing the average length of hospitalisation, mortality, antibiotic consumption, and antibiotic expenditure [7–11].

The effort to initiate AMS has led to the establishment of a multidisciplinary AMS team in many hospitals in Malaysia. The concerns over possible adverse clinical outcomes such as poor clinical response and mortality are postulated to be the major hindrance in implementing of AMS activities. However, investigations on the acceptance of AMS interventions and their impact on clinical outcomes among hospitalised patients, especially in Malaysia, are lacking.

Therefore, this study aimed to determine the acceptance of carbapenem stewardship interventions, its impact on clinical outcomes and the factors affecting the 30-day mortality among patients on carbapenem.

## Methods

### Design, setting, and population

This retrospective cohort study utilised the routinely collected data extracted from AMS review forms and patient medical records. The population studied was hospitalised adult (≥ 18 years old) patients in the medical wards of Kuala Lumpur Hospital, the largest tertiary hospital in Malaysia. Patients initiated on carbapenem (intravenous meropenem, imipenem/cilastatin or ertapenem) by the primary team either as prophylaxis, empirical or microbiological confirmed therapy; reviewed by AMS team and classified as non-justified use of carbapenem were included in the study. Cases initiated by other disciplines referred to the infectious disease team or reviewed after seven days on carbapenem were excluded.

### Carbapenem stewardship review

The carbapenem stewardship review was conducted by a multidisciplinary AMS team consisting of infectious diseases consultants/specialists, pharmacists, microbiologists, and infection control nurses. The reviews were conducted weekly in all medical wards, in which patients initiated on carbapenems were identified and reviewed. Upon review, the AMS team determined the judiciousness of carbapenem initiation, subsequently classified into justified and non-justified use of carbapenems. Unjustified use of carbapenems refers to the use of carbapenems when it is not indicated, where appropriate and adequate coverage (optimal dose and duration) and cost-effective therapy for the diagnosis or suspected infection were not provided or given [12]. As for non-justified use, the prescribing problem and its cause/s were identified, and interventions were recommended. Subsequently, the primary team's physician decided whether to accept or decline the intervention. All information, including the prescribing problems, causes, interventions, acceptance and outcomes, were recorded in the AMS review form.

### Data collection

A list of patients on carbapenem in the medical wards and reviewed by the AMS team from 1^st^ January 2016 to 31^st^ December 2019 was obtained from the AMS census. A stratified random sampling method was applied where subjects were sampled from all cases fulfilling the eligibility criteria. The cases were stratified into two groups based on the acceptance of the interventions by the primary team and labelled as the accepted and not-accepted groups. A random sampling method was used to achieve each group's required sample size. Patient comorbidities were assessed using the Charlson Comorbidity Index (CCI), a sum of scores based on the weightage of each disease towards the risk of mortality. The higher the CCI, the higher the chance of mortality [13].

### Measured outcomes

The measures were classified based on the PCNE drug-related problem classification Version 8.01 [14]. The measured outcomes include AMS interventions and the acceptance status, clinical outcomes at day-7 and day-30, and predictors of 30-day mortality. Clinical outcome at day-7 includes total white blood cells (TWBC), C-reactive protein (CRP) and body temperature. Clinical outcome at day-30 includes patient's conditions such as being discharged well, hospitalised with improving health, hospitalised with deteriorating health, or deceased within thirty days from the AMS intervention.

### Sample size and statistical analyses

The sample size was calculated using the Fleiss formula with 80% study power and 95% confidence level [15]. The sample size ratio was set at 0.36, with the percentage of accepted and not-accepted interventions of 50% and 22%, respectively, based on our preliminary data in 2016 [16]. A minimum of 136 patients (100 in the accepted group and 36 in the not-accepted group) was required to fulfil the sample size need.

Data analyses were conducted using IBM® SPSS® Statistics Version 24. Categorical data were compared using Chi-square or Fisher's exact test and reported in frequency and percentage (%). The distribution of patients' age was normally distributed and compared between groups using an independent t-test. The length of hospital stay was not normally distributed and was compared with Mann–Whitney Rank U test. Multiple logistic regression forward LR was used to determine the predictors of 30-day mortality. Variables with p < 0.25 in binary logistic regression were included in the final model, where multicollinearity, interactions between variables, model fitness, classification table and ROC curve tests were also performed. Statistical significance was set at p < 0.05.

## Results

### Patient demographic and baseline characteristics

**Fig. 1:**
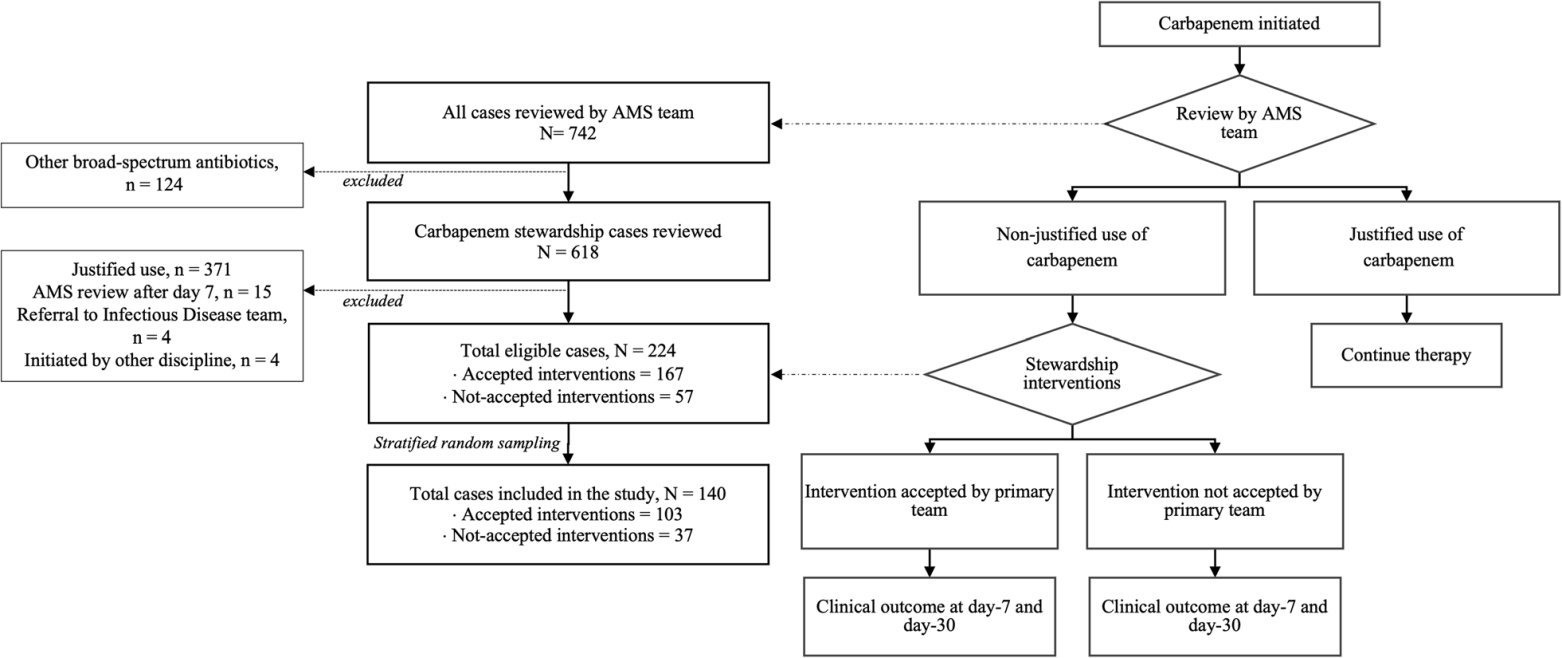
Carbapenem stewardship review flow and patient selection for the study

During the study period, 742 cases of broad-spectrum antibiotics were reviewed under the hospital's AMS program (Fig. 1). A total of 140 cases were included in this study, consisting of 103 accepted and 37 not-accepted intervention cases (ratio of 0.36). Patient demographics and clinical characteristics are summarised in Table 1. The mean age of patients was 57.6 (SD = 18.3) years, with an almost equal distribution of males (48.6%) and females (51.4%). The CCI was similar between the groups, and 55.0% had a CCI of more than three. A fraction of 37 (26.4%) subjects had a history of hospitalisation in the last three months, with 8% having prior ICU admission. Although 20 subjects (14.3%) had prior broad-spectrum antibiotic exposure, only six (4.3%) had MRO infection. Nevertheless, the exposure to broad-spectrum antibiotics over the past three months was significantly different between the groups; 11 (10.7%) were in the accepted and 9 (24.3%) were in the not-accepted groups (p = 0.042).

**Table 1:** Patient demographic and clinical characteristics before carbapenem initiation

Characteristics	Total n (%)	Accepted n (%)	Not-accepted n (%)	p-value
Age (years old)				0.097^a^^*^
Mean (SD)	57.6 (18.3)	59.4 (16.1)	52.5 (22.9)	
Gender				0.709
Female	72 (51.4)	52 (50.5)	20 (54.1)	
Male	68 (48.6)	51 (49.5)	17 (45.9)	
Race				0.504
Malay	77 (55.0)	56 (54.4)	21 (56.8)	
Chinese	26 (18.6)	17 (16.5)	9 (24.3)	
Indian	34 (24.3)	28 (27.2)	6 (16.2)	
Others	3 (2.1)	2 (1.9)	1 (2.7)	
Comorbidities				
Diabetes mellitus	67 (47.9)	56 (54.4)	11 (29.7)	0.010^*^
Hypertension	51 (36.4)	44 (42.7)	7 (18.9)	0.010^*^
Renal disease	27 (19.3)	18 (17.5)	9 (24.3)	0.365
Stroke	24 (17.1)	23 (22.3)	1 (2.7)	0.007^*^
History of myocardial infarction	23 (16.4)	15 (14.6)	8 (21.6)	0.320
Congestive heart failure	20 (14.3)	13 (12.6)	7 (18.9)	0.348
Connective tissue disease	16 (11.4)	7 (6.8)	9 (24.3)	0.012^b*^
Charlson comorbidity index (CCI)				
≤ 3	63 (45.0)	43 (41.7)	20 (54.1)	0.197
> 3	77 (55.0)	60 (58.3)	17 (45.9)	
Hospitalisation within last 90 days				
Yes	37 (26.4)	26 (25.2)	11 (29.7)	0.595
No	103 (73.6)	77 (74.8)	26 (70.3)	
ICU admission within last 90 days				
Yes	11 (7.9)	5 (4.9)	6 (16.2)	0.067^b^
No	129 (92.1)	98 (95.1)	31 (83.8)	
MRO infection within last 90 days				
Yes	6 (4.3)	3 (2.9)	3 (8.1)	0.188^b^
No	134 (95.7)	100 (97.1)	34 (919)	
Broad-spectrum antibiotic use within last 90 days				
Yes	20 (14.3)	11 (10.7)	9 (24.3)	0.042^*^
No	120 (85.7)	92 (89.3)	28 (75.7)	
Site of infection				0.283^b^
Respiratory	41 (29.3)	29 (28.2)	12 (32.4)	
Urinary tract	19 (13.6)	16 (15.5)	3 (8.1)	
Hepatobiliary	18 (12.9)	10 (9.7)	8 (21.6)	
Skin and soft tissue	13 (9.3)	12 (11.7)	1 (2.7)	
Blood stream	13 (9.3)	9 (8.7)	4 (10.8)	
Central nervous system	5 (3.6)	5 (4.9)	0 (0.0)	
Intra-abdominal	3 (2.1)	2 (1.9)	1 (2.7)	
Multiple foci	3 (2.1)	3 (2.9)	0 (0.0)	
Unknown source	25 (17.5)	17 (16.5)	8 (21.6	
Organisms isolated				0.472^b^
No organism isolated	81 (57.9)	57 (55.3)	24 (64.9)	
*K.pneumoniae* (ESBL)	16 (11.4)	14 (13.6)	2 (5.4)	
*K.pneumoniae* (non-ESBL)	8 (5.7)	6 (5.8)	2 (5.4)	
*K.pneumoniae* (CRE)	1 (0.7)	1 (1.0)	0 (0.0)	
*E.coli* (non-ESBL)	7 (5.0)	6 (5.8)	1 (2.7)	
*E.coli* (ESBL)	4 (2.9)	3 (2.9)	1 (2.7)	
*P.aeruginosa*	2 (1.4)	1 (1.0)	1 (2.7)	
*P.aeruginosa* (MRO)	2 (1.4)	0 (0.0)	2 (5.4)	
*Proteus spp.* (ESBL)	2 (1.4)	2 (1.9)	0 (0.0)	
*Proteus spp*. (non-ESBL)	2 (1.4)	2 (1.9)	0 (0.0)	
*Morganella morganii*	2 (1.4)	2 (1.9)	0 (0.0)	
Gram positive	2 (1.4)	1 (1.0)	1 (2.7)	
*A.baumanii* (MRO)	2 (1.4)	2 (1.9)	0 (0.0)	
*Burkolderia pseudomallei*	1 (0.7)	1 (1.0)	1 (1.0)	
*Citrobacter spp.* (ESBL)	1 (0.7)	1 (1.0)	0 (0.0)	
Mixed growth	6 (4.3)	4 (3.9)	2 (5.4)	
Presence of bacteraemia	23 (16.4)	18 (17.5)	5 (13.5)	0.577
Presence of sepsis/septic shock	44 (31.4)	30 (29.1)	14 (37.8)	0.328

Chi-square test was performed unless otherwise stated

^a^Independent-samples T-test. ^b^Fisher’s exact test

^*^p<0.05 denotes statistical significance

Meropenem was the commonly prescribed carbapenem (68.6%), and empirical therapy was initiated for 64.3% of the patients (60.2% and 75.7% cases from the accepted and not-accepted groups, respectively), while the remaining were culture-directed. More than half of the cultures taken had no significant bacterial growth (57.9%), and 4.3% grew mixed growth of organisms. Twenty-three (16.4%) of the total isolates were extended-spectrum beta-lactamases producers (ESBL). They were isolated from non-sterile sites such as tracheal aspirate/ sputum, pus, bed-site tissue samples or urine. The most commonly isolated organisms were *Klebsiella pneumoniae* (17.8%), where 11.4% were ESBL, 5.7% were sensitive, and one (0.7%) was CRE. Non-ESBL organisms caused 23 (16.4%) cases of bacteraemia. A total of 44 study cases (31.4%) were diagnosed with sepsis or septic shock before carbapenem initiation.

### AMS interventions and acceptance

The most frequently encountered drug-related problems were unnecessary carbapenem treatment (97.9%; Table 2). Most carbapenem prescriptions were categorised as having no clear indication (55%) or inappropriate according to the recommendation by local antibiotic guidelines (45%). Discontinuation of antibiotics therapy was recommended for all cases with no clear indication for antibiotic use. Escalation to colistin was recommended in three cases of therapy failure or inadequate effect with the use of carbapenem due to the presence of carbapenem-resistant infections. There was no significant difference in the acceptance status between types of interventions (p > 0.05). More than 54% (n = 20) of the non-acceptance of AMS interventions were due to the primary physician's opinion that the patient's clinical condition was deemed unsuitable for de-escalation. The physician's intention to complete the currently prescribed antibiotic for one week was documented in 10 (27.0%) cases. Other reasons for non-acceptance were the patients deemed clinically responded despite no positive culture, n = 6 (16.2%) or despite culture results showing resistance, n = 1 (2.7%).

**Table 2:** Types of carbapenem drug-related problems identified, its causes and interventions made

	Total n (%)	Accepted n (%)	Not-accepted n (%)	p-value
Drug-related problems (DRP)				0.784^b^
Unnecessary antibiotic treatment	137 (97.9)	101 (73.7)	36 (26.3)	
No effect or therapy failure	3 (2.1)	2 (66.7)	1 (33.3)	
Causes of DRP				0.802^a^
No indication for carbapenem use	77 (55.0)	56 (72.7)	21 (27.3)	
Inappropriate antibiotic choice according to the guideline	63 (45.0)	47 (74.6)	16 (25.4)	
Types of interventions				0.136^b^
Discontinuation	70 (50.0)	47 (67.1)	23 (32.9)	
De-escalation	67 (47.9)	54 (80.6)	13 (19.4)	
Escalation	3 (2.1)	2 (66.7)	1 (33.3)	

^a^Chi-square test. ^b^Fisher’s exact test

### Clinical outcomes

The day-7 and day-30 clinical outcomes and length of hospital stay are shown in Table 3. Overall, most patients became afebrile and showed decreased or normalised TWBC and CRP at day-7, but the differences between the groups' measures were non-significant. Overall outcome at day-30 between the groups did not differ significantly. The mortality at day-30 in the accepted and not-accepted groups were 32% and 35%, respectively (p = 0.731). The discharge rate was also comparable between the groups (53.4% *versus* 45.9%, p = 0.437). The median (IQR) length of hospitalisation in the accepted group was noted at 36.0 (23.5 – 48.5) versus the not accepted group at 30.0 (11.5 – 48.5) days (p = 0.526).

**Table 3:** Clinical outcomes at day-seven and day-thirty

Outcomes	Total n (%)	Accepted n (%)	Not-accepted n (%)	p-value
Outcomes at day-7				
TWBC trend				0.578^a^
Decreasing or normalised	114 (81.4)	85 (74.6)	29 (25.4)	
Increasing or elevated	26 (18.6)	18 (69.2)	8 (30.8)	
CRP trend^d^				0.533^b^
Decreasing or normalised	52 (75.3)	39 (75.0)	13 (25.4)	
Increasing or elevated	17 (24.6)	11 (64.7)	6 (35.3)	
Temperature				0.164^a^
Afebrile	113 (80.7)	86 (76.1)	27 (23.9)	
Febrile	27 (19.3)	17 (63.0)	10 (37.0)	
Outcome at day-30				
Discharged well	72 (51.4)	55 (76.4)	17 (23.6)	0.568^b^
Mortality	46 (32.9)	33 (71.7)	13 (28.3)	
Still hospitalised (ill)	12 (8.6)	7 (58.3)	5 (41.7)	
Still hospitalised (improving)	10 (7.1)	8 (80.0)	2 (20.0)	
Length of stay (days)				0.526^c^
Median (IQR)	34.5 (30.5–48.5)	36.0 (23.5–48.5)	30.0 (11.5–48.5)	

Normalising or decreasing trend: TWBC approaching 4.0-11.0 x 10^9^/L or CRP approaching <10.0mg/L

Increasing or elevated trends: TWBC increasing above 11.0 × 10^9^/L and CRP >10mg/L.

Febrile: temperature >37 °C, afebrile: temperature =37 °C

^a^Chi-square test. ^b^Fisher’s exact test. ^c^Mann Whitney test

^d^N = 69 as repeated CRP levels were not available for the remaining 71 cases

### Predictors of 30-day mortality

Among patients with unjustified carbapenem use (Table 4), CCI with a score of more than three increased the odds of mortality by almost three times (OR = 2.84, 95% CI = 1.28–6.29, p = 0.010), and febrile at day-7 increased the odds of mortality by 4.5 times (OR = 4.58, 95% CI = 1.83–11, p = 0.010). Other factors, including the intervention acceptance status, did not significantly predict mortality.

**Table 4:** The predictors of thirty-day mortality

Variables	Binary Logistic Regression		Multiple Logistics Regression^d^	
	OR^b^ (95% CI)	p value	OR^c^ (95% CI)	p value
CCI > 3	2.49 (1.18, 5.25)	0.017^a^	2.84 (1.28, 6.29)	0.010
Febrile at day-7	4.02 (1.68, 9.64)	0.002^a^	4.58 (1.83, 11.5)	0.001
Increasing or elevated TWBC at day-7	2.46 (1.03, 5.85)	0.043^a^		
Sepsis at baseline	1.94 (0.92, 4.09)	0.080^a^		
Acceptance of intervention	0.87 (0.39, 1.92)	0.731		
Bacteraemia at baseline	1.14 (0.43, 3.01)	0.787		
Previous ICU admission	1.18 (0.33, 4.27)	0.797		
Previous broad-spectrum antibiotics use	0.86 (0.31, 2.40)	0.769		
Previous hospitalisation	0.97 (0.44, 2.17)	0.949		
Previous MRO infection	1.02 (0.18, 5.80)	0.980		

No interactions and multi-collinearity detected. Correlation matrix = 0.183

Model is fit; Hosmer–Lemeshow test (χ^2^ = 0.001, df = 2, p = 0.999)

Classification table (overall correctly classified = 71.4%)

Area under ROC curve = 0.690 (95% CI 0.560, 0.78; p < 0.001)

^a^Variables included in the multiple logistic regression

OR^b^: Crude odds-radio

OR^c^: Adjusted odds-ratio

CI: Confidence interval

^d^Final model after forward LR method applied (excluding sepsis and TWBC trend)

## Discussion

Discontinuation, de-escalation, and dosage optimisation of antibiotics are the common AMS interventions. This study found that de-escalation may be the preferred AMS intervention, as it has the highest acceptance rate, although discontinuation was mostly suggested. Similarly, Seah et al. reported discontinuation of carbapenem as their main AMS intervention, followed by de-escalation and optimisation of dosing. They reported that the acceptance of interventions to de-escalate is higher than discontinuation, which is comparable to this study[10]. This is supported by another study, where the percentage of acceptance of interventions for de-escalation was double the acceptance of recommendations to stop carbapenems [9]. The better acceptance for de-escalation may be due to the physicians feeling more justified of having at least a narrow-spectrum antibiotic coverage rather than no antibiotics for their patients.

Favourable clinical outcome or response at day-7 are defined as decreasing or normalised TWBC and CRP levels; and being afebrile. This study found a higher percentage of patients in the intervention accepted group to have favourable clinical outcomes at day-7, although the differences were not statistically significant. These outcomes are comparable to studies which showed similar clinical success at day-7 between the accepted and not-accepted groups [8–10]. This observation may support that AMS intervention did not worsen the markers for infection and may dismiss the notion that discontinuation or de-escalation of antibiotics may worsen the patient conditions [17].

Clinical outcomes at day-30 represented by the discharge and mortality rate were also similar between the accepted and not-accepted groups. These findings are also comparable to other published studies, which showed similar survival rates at discharge [8, 9, 18] and mortality [8, 17, 18]. These findings suggest that a thorough and systematic assessment carried out prior to AMS intervention may provide low-risk complications of discontinuation or de-escalation of antibiotics. There was no significant reduction in length of hospitalisation between groups in this study, similar to other studies in the region [8–10]. This may be due to the direct effect of AMS interventions on antimicrobial therapy alone and not the mainstay patient management plan. Therefore, the changes in antimicrobial therapy may not significantly affect the hospital stays of patients with primary medical conditions not relating to infection.

The study also found no significant association between acceptance of AMS intervention and mortality. Most studies which investigated the impact of accepting AMS interventions on mortality found that there was no significant association between acceptance and mortality [10, 11, 19]. However, Teng et al. reported that non-acceptance of AMS recommendation was associated with almost three times increase in 30-day mortality risk [9]. This study found two independent predictors for 30-day mortality among patients with unjustified use of carbapenem; CCI and body temperature > 37 °C at day-7. Although there are studies reporting CCI as a significant predictor of mortality, none however reported on body temperature. Okumura et al. reported increased hazard ratio with increasing CCI among patients in general wards and intensive care unit, while Palacios-Baena et al. found an increased hazard of mortality with CCI > 3 among patients with Enterobacterales bacteraemia [11, 20]. Apart from that, this study also found that being febrile at day-7 has almost 4.5 times the odds of mortality compared with those who are afebrile. No study reported on body temperature as a significant predictor of mortality in stewardship programs.

Fever is commonly associated with lower mortality risk in septic patients. A meta-analysis showed that the mortality rate among normothermic and hypothermic septic patients was significantly higher than that of febrile patients [21]. However, one study reported that temperature ≥ 38.5 °C was significantly associated with increased mortality in non-septic patients [22]. In addition, fever can also occur due to non-infective causes such as drug-induced fever, thromboembolism, brain injury, pancreatitis, autoimmune diseases, malignancy and endocrine disorders [23]. Since the current study consists of more patients without sepsis or clear indications of infection, being febrile can signify other underlying conditions that may predispose to mortality.

The current study has highlighted several significant findings to support the feasibility of AMS interventions. The outcome of this study can be more widely applied to further enforce and promote AMS. It can be used to reinforce the positive impacts of accepting or implementing AMS strategies without the increased concern over any negative impact on the patient’s clinical outcome. Evidence on the clinical implications of AMS in this study can be used to further aid in the development and implementation of hospital-level AMS policy so that AMS initiatives can be expanded to other than medical disciplines as well.

Nevertheless, some limitations may have influenced the findings of this study. The nature of retrospective study design has methodological limitations such as incomplete or missing data. The secondary data is collected based on the information found on the AMS review forms and patients' medical records. CRP levels were not available for all patients included in the study. However, the extracted information from the existing data was sufficient for this study's analysis. This study did not include other impacts such as adverse events and investigation into the emergence of resistant organisms.

## Conclusion

The acceptance of the antibiotic stewardship interventions was good, where de-escalation was the preferred intervention among the primary physicians. The clinical outcomes at day-7 and day-thirty post-AMS interventions were similar regardless of physicians' acceptance status. No negative impact was observed in accepting stewardship interventions. This encourages more acceptance of future AMS interventions and activities.

## Data Availability

All data generated and/or analysed during the current study are included in this article. The datasets used and/or analysed during the current study are available from the corresponding author on reasonable request.

## Abbreviations

AMS: Antimicrobial stewardship
CCI: Charlson Comorbidity Index
CI: Confidence interval
CRE: Carbapenem-resistant Enterobacterales
CRP: C-reactive protein
DRP: Drug-realted problem
ESBL: Extended-spectrum beta-lactamases producers
ICU: Intensive care unit
IQR: Interquartile range
MRO: Multi-drug resistant organisms
OR: Odds ratio
SD: Standard deviation
TWBC: Total white blood cells
WHO: World Health Organization
